# 314. Ceftazidime-Avibactam Versus Meropenem-Vaborbactam for the Treatment of Carbapenem-Resistant Enterobacterales Infections

**DOI:** 10.1093/ofid/ofae631.104

**Published:** 2025-01-29

**Authors:** Sara M Karaba, Dariusz A Hareza, Armani Hawes, Yehudit Bergman, Suiyini Fiawoo, Jae Hyoung Lee, Sara E Cosgrove, Patricia J Simner, Pranita Tamma

**Affiliations:** Johns Hopkins University, Baltimore, MD; Johns Hopkins University, Baltimore, MD; Johns Hopkins Hospital, Baltimore, Maryland; Johns Hopkins, Baltimore, Maryland; Johns Hopkins School of Medicine, Baltimore, Maryland; Johns Hopkins, Baltimore, Maryland; Johns Hopkins School of Medicine, Baltimore, Maryland; Johns Hopkins School of Medicine, Baltimore, Maryland; Johns Hopkins School of Medicine, Baltimore, Maryland

## Abstract

**Background:**

Infections due to carbapenem-resistant Enterobacterales (CRE) are increasing in the United States, most commonly the result of *Klebsiella pneumoniae* carbapenemase (KPC) production. Both ceftazidime-avibactam (CZA) and meropenem-vaborbactam (MVB) are preferred treatment options for CRE infections but comparative effectiveness data to assist clinicians with preferentially selecting between these agents are limited.

**Figure:**
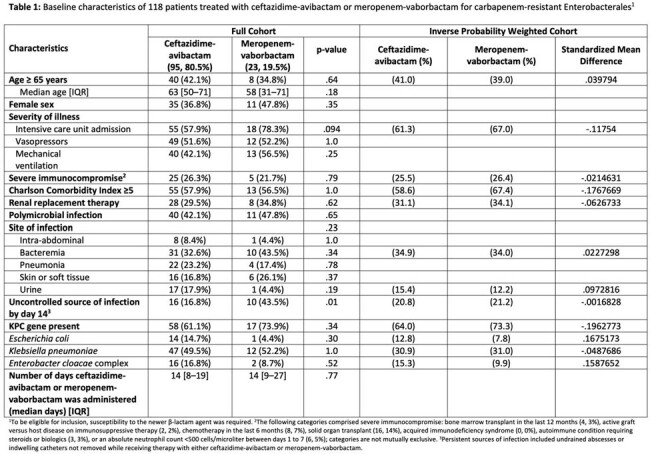

Baseline characteristics of 118 patients treated with ceftazidime-avibactam or meropenem-vaborbactam for carbapenem-resistant Enterobacterales

**Methods:**

We conducted an observational study of patients with CRE infections between 2018–2023 who received ≥ 3 days of CZA or MVB across five Maryland hospitals. Susceptibility to the administered agent (i.e., CZA or MVB) was required for eligibility. Inverse probability weighting (IPW) using propensity scores was used to assess the primary outcome of 30-day mortality. Development of resistance (i.e., ≥ 4-fold increase in MIC of the agent used to treat the index infection) within 90 days assessed by broth microdilution was a secondary outcome.

**Figure:**
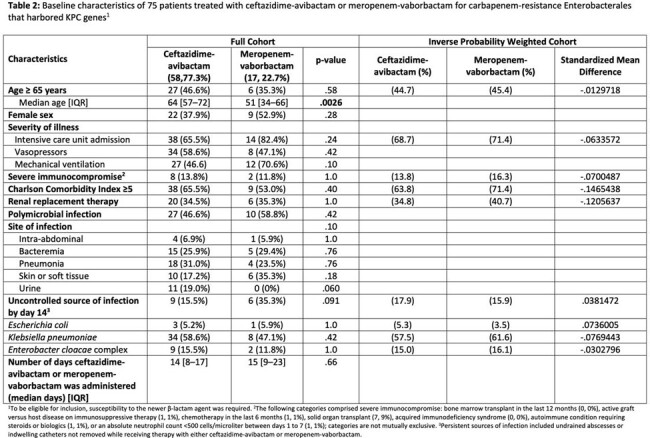

Baseline characteristics of 75 patients treated with ceftazidime-avibactam or meropenem-vaborbactam for carbapenem-resistance Enterobacterales that harbored KPC genes

**Results:**

In the entire cohort there were 95 and 23 patients in the CZA and MVB groups, respectively. Balance was achieved across all variables in the IPW cohort (Table 1). 30-day mortality was similar between the antibiotic groups comparing CZA to MVB (aOR 0.997, 95% CI 0.86–1.63, p=0.97) and in the subgroup of 75 patients with isolates with an identified *bla*_KPC_ gene present who underwent IPW with good balance achieved (Table 2) (aOR 1.02, 95% CI 0.85–1.21, p=0.87).

**Conclusion:**

In a cohort of patients with CRE infections treated with either CZA or MVB, 30-day mortality was similar. The frequency of subsequent emergence of resistance is currently being investigated.

**Disclosures:**

**Sara M. Karaba, MD, PhD, MHS**, Entasis: Advisor/Consultant **Patricia J. Simner, PhD**, Affinity Biosensors: Grant/Research Support|BD Diagnostics: Grant/Research Support|bioMérieux: Grant/Research Support|Entasis: Personal fees|GeneCapture: Personal Fees|Merck: Personal fees|OpGen Inc: Grant/Research Support|Qiagen: Grant/Research Support|Shionogi: Personal fees|T2 Biosystems: Grant/Research Support

